# Dedicated teams to optimize quality and safety of surgery: A systematic review

**DOI:** 10.1093/intqhc/mzac078

**Published:** 2022-10-06

**Authors:** C M Lentz, R A F De Lind Van Wijngaarden, F Willeboordse, L Hooft, M J van der Laan

**Affiliations:** Department of Surgery (Division of Vascular Surgery), University Medical Center Groningen, Hanzeplein 1, Groningen 9713 GZ, The Netherlands; Department of Cardiothoracic Surgery, Leiden University Medical Center, Albinusdreef 2, Leiden 2333 ZA, The Netherlands; Department of Cardiothoracic Surgery, Amsterdam University Medical Center, Meibergdreef 9, Amsterdam 1105 AZ, The Netherlands; Knowledge Institute of Medical Specialists, Mercatorlaan 1200, Utrecht 3528 BL, The Netherlands; Cochrane Netherlands/Julius Center for Health Sciences and Primary Care, University, Utrecht Medical Center, Heidelberglaan 100, Utrecht, 3584 CX, The Netherlands; Department of Surgery (Division of Vascular Surgery), University Medical Center Groningen, Hanzeplein 1, Groningen 9713 GZ, The Netherlands

**Keywords:** dedicated team, team training, operative process, quality, safety, surgery

## Abstract

**Background:**

A dedicated operating team is defined as a surgical team consisting of the same group of people working together over time, optimally attuned in both technical and/or communicative aspects. This can be achieved through technical and/or communicative training in a team setting. A dedicated surgical team may contribute to the optimization of healthcare quality and patient safety within the perioperative period.

**Method:**

A systematic review was conducted to evaluate the effects of a dedicated surgical team on clinical and performance outcomes. MEDLINE and Embase were searched on 23 June 2022. Both randomized controlled trials (RCTs) and non-randomized studies (NRSs) were included. Primary outcomes were mortality, complications and readmissions. Secondary outcomes were costs and performance measures.

**Results:**

Fourteen studies were included (RCTs *n* = 1; NRSs *n* = 13). Implementation of dedicated operating teams was associated with improvements in mortality, turnover time, teamwork, communication and costs. No significant differences were observed in readmission rates and length of hospital stay. Results regarding duration, glitch counts and complications of surgery were inconclusive. Limitations include study conduct and heterogeneity between studies.

**Conclusions:**

The institution of surgical teams who followed communicative and/or technical training appeared to have beneficial effects on several clinical outcome measures. Dedicated teams provide a feasible way of improving healthcare quality and patient safety. A dose–response effect of team training was reported, but also a relapse rate, suggesting that repetitive training is of major concern to high-quality patient care. Further studies are needed to confirm these findings, due to limited level of evidence in current literature.

**Prospero registration number:**

CRD42020145288

## Introduction

An essential part of incidents in healthcare takes place in the perioperative process. The complexity of technical, logistical and communicative interactions creates a high-risk environment for patients [1]. The multitude of players and handovers in this process make it vulnerable for mistakes, information loss and communication errors [2, 3]. Moreover, the operative process is constantly changing. Perpetual improvement and adjustment are mandatory to ensure patient safety [1].

Increasing complexity of surgical procedures and eligible patient populations has led to medical specialists super specializing within their field of expertise. This extensive specialization is not always matched by other members of the operating room (OR) team, i.e. scrub and circulating nurses and anesthesiologists. In many cases, team composition changes frequently. When there is a progressive mismatch between super specialists and generalists within one team, it is at risk of communication problems, mismatch of perioperative expectations and non-alignment of the appreciation of the perioperative risk. It seems straightforward that a dedicated surgical team, trained in technical and/or nontechnical skills concerning an intervention, could improve the efficiency, quality and safety of healthcare. However, knowledge regarding the impact of a dedicated surgical team on surgical outcomes is still lacking.

A clear definition of a dedicated surgical team is still lacking. Relevant literature focuses on either technical training or pure nontechnical skill training. In our opinion, a true dedicated team is trained in both technical and nontechnical skills, as a team. In this review, a dedicated surgical team is defined as a surgical team consisting of the same group of people working together over time, optimally attuned in technical and/or communicative aspects, ideally both. This can be achieved through communicative and technical training in a team setting. Team composition and team training are essential components of a dedicated team; however, with varying team compositions being frequent, especially in larger medical centers, team training is considered the most important aspect of a dedicated team.

Previous literature focused on nontechnical skills training for surgical teams found communication and teamwork to improve following training, with effects remaining visible for a varying period [4–7].

Team training was also found to enable the cultivation of a shared mental model; that is where surgical staff have a mutual awareness regarding the intricacies of the operation and clear allocated tasks and roles [8]. Team familiarity, which can establish a shared mental model, has been found to have a positive impact on performance including surgical time reduction [8].

The most extensively studied training is crew resource management (CRM) for OR teams [9, 10]. CRM is adapted from aviation aiming to reduce human errors in high-stakes environments [9, 10]. Nonetheless, CRM training is not uniform across studies; there are essential differences in what is incorporated in CRM and the recommended frequency of provided trainings [10, 11]. In all cases, the first step should involve defining the problem that needs to be addressed during training. An exact purpose of the intervention should be established; providing CRM is not a goal on itself. The selection of clinically relevant measures to monitor outcomes of the intervention poses a challenge [10]. In most cases, complications or mortality are confounded by several factors and hence potentially inaccurate measures in a complex environment.

The aim of this review was to determine whether a dedicated surgical team trained in communicative and/or technical skills contributes to the optimization of healthcare quality and patient safety within the perioperative period. Clinical outcomes, performance measures and costs were considered to establish whether dedicated surgical teams result in superior outcomes compared to non-dedicated surgical teams.

## Methods

### Patient and public involvement

No patients were involved in this study.

### Definitions

In this study, a dedicated surgical team was defined as a surgical team, optimally attuned in technical and/or communicative aspects. Teambuilding could be performed through communicative and technical training in a team setting. A dedicated surgical team includes surgeon, scrub and circulating nurses, anesthesiologist, nurse anesthetist and may include additional members (i.e. perfusionists).

Glitch counts refer to disturbances during surgery and are expressed as number of glitches per surgery per hour. Turnover time refers to time from when a patient leaves the surgical room until another patient enters the same surgical room.

### Search strategy and selection criteria

The MEDLINE (via Ovid) and Embase (via Embase.com) databases were searched on 23 June 2022. Relevant search terms for operations and dedicated teams were used, in combination with search terms regarding clinical outcomes or safety (supplementary file 1). PICOS (Population, Intervention, Control and Outcomes) criteria are shown in supplementary file 3. Clinical outcomes included mortality, complications and readmissions. Secondary outcomes were costs and performance measures, including length of hospital stay, glitch count, operating time, turnover time, teamwork and communication.

Studies were selected, based on the following selection criteria: conducted after the year 2000, randomized controlled trials (RCTs) or non-randomized comparative studies (NRSs; including before-and-after studies) comparing outcomes following the use of a dedicated surgical team versus a non-dedicated surgical team, a sample size of at least 20 surgeries, conducted in the Western world and reporting at least one clinical outcome parameter (mortality, postoperative complications and readmission rate). These selection criteria were chosen to ensure that studies were conducted in comparable settings to those seen in Dutch surgical clinics, allowing for translation of results into practice.

### Selection procedure

Papers retrieved during the database searches were selected by a two-step screening process. A dual independent review of the search results, based on title and abstract, was conducted by two reviewers (M.L. and R.L.W.). During the second step, full-text evaluation was performed by the same two reviewers. Discrepancies in the study selection were resolved by group discussion (M.L., R.L.W. and F.W.).

### Risk of bias

For determining the risk of bias in the RCTs, an adjusted version of the Cochrane Risk of Bias Tool was used and for NRSs, the Newcastle–Ottawa tool (see Tables 3–4) was used.

## Results

### Selected papers

The search yielded 890 records (after duplicate removal). After a review based on title and abstract, 116 studies were selected for full paper evaluation. After this evaluation, 106 more studies were excluded. Reasons for exclusion included not meeting the research question, lack of clinical outcomes, non-comparative studies and not being an original study (see the Preferred Reporting Items for Systematic Reviews and Meta-Analyses flowchart, supplementary file 2). Additional sources, obtained by reference checking from selected studies, yielded four extra eligible studies.

Fourteen studies were included in this systematic review (RCTs *n* = 1; NRSs *n* = 13). The most important characteristics and results are included in the evidence tables (Tables 1 and 2). The intervention entails team training to form a dedicated team. The approach, duration and frequency of team training vary across studies. Setting, intervention type and outcomes are largely heterogeneous, making it difficult to pool or summarize studies. Results were therefore descriptive; conclusions were drawn where possible.

**Table 1:** Characteristics of the included ‘dedicated teams’ studies

Study	Setting	Intervention	Outcomes	Study information
[21]	Total joint arthroplasty	Dedicate efficient teams Parallel processes Dedicated hospital resources Adjustment behavior physicians	Turnover time Length of stay	Uncontrolled before-and-after study Measurement after 3 years Number of surgeries = unknown
[15]	Robotic-assisted sacrocolpopexy (RASC)	Dedicated Robotic Team (by one surgeon)	Complications Readmissions Duration of surgery Length of stay	Retrospective cohort study Data during admission Intervention: *n* = 17 Control: *n* = 71
[14]	Surgery (orthopedics, urology, gynecology, digestive, cardiovascular, neurosurgery)	CRM team training	Mortality Major complications Postoperative complications	Cluster RCT Follow-up outcomes 30 days Intervention: 8623 Control: 8222
[20]	Elective orthopedic surgery	Parallel processes Standardized protocols for spinal anesthesia and perioperative patient management Team training	Complications Duration of surgery Costs	Controlled before-and-after study Intervention: 78 Control: 89
[12]	All types of surgery	TeamSTEPPS program for all staff	Mortality Morbidity Turnover time Teamwork Communication	Retrospective cohort study Measurements 9 and 21 months after the intervention Number of surgeries unknown
[22]	Acute care (bile ducts)	Acute care surgery model, dedicated operation team with one dedicated operating theater daytime	Duration of surgery Length of stay	Prospective cohort study Data during admission for 2 years after implementation Intervention: 72 Control: 172
[25]	Cardiothoracic, neuro- and orthopedic surgery	Interprofessional education initiative	Turnover time Costs	Uncontrolled before-and-after Data collection for 12 months after the start of the initiative 50 ORs
[23]	Elective orthopedic plastic or vascular surgery	Safety-improvement program one of more elements: Teamwork training Restructured systems and standardization (SOP) Lean quality improvement	Glitch count NOTECHS II (nontechnical skills)	5 identical controlled before-and-after studies with the analysis of pooled data Data during admission 3 months after intervention All interventions: 255 Control: 198 Pre-intervention: 219 Post-intervention: 234
[16]	Laparoscopic cholecystectomy and carotid endarterectomy	CRM training and coaching	Complications Technical operative mistakes Duration of surgery Length of stay NOTECHS II SAQ teamwork climate	Uncontrolled before-and-after study Data during admission 3 months after intervention Pre-intervention: 48 Post-intervention: 55
[19]	Elective orthopedic surgery	SOPs and team training based on CRM	Complications Readmissions Glitch rate Length of stay NOTECHS II	Controlled before-and-after study Data during admission and 3 and 6 months after the intervention Pre-intervention *n* = 567 (30 observations) Post-intervention: 679 (33 observations) Pre-control: n = 544 (17 observations) Post-control: *n* = 421 (21 observations)
[17]	Elective orthopedic and vascular/general surgery	CRM team training + coaching	Complications Readmissions Glitch rate Length of stay Duration of surgery NOTECHS II	Uncontrolled before-and-after study Data during admission 3 months after the intervention Pre-intervention: *n* = 26 Post-intervention: *n* = 25 Pre-control *n* = 11 Post-control n = 10
[13]	Noncardiac surgery	Team training + coaching	Mortality	Retrospective cohort study with control Training in 74 institutions Total: 182 409 operations
[24]	Robot-assisted laparoscopic radical prostatectomy for prostate cancer	Trainee adherence vs time-oriented surgical goals Dedicated anesthesiology team Simultaneous processes by nurses, urologists during turnover Identification and elimination of unused disposables	Duration of surgery Anesthesiology time Turnover time Costs	Before-and-after study Data during admission 8 months after the intervention Pre: *n* = 100 Post: *n* = 100
[18]	Elective orthopedic (control) and plastic surgery (intervention)	Teamwork training and lean process improvement intervention	Complications Readmissions Length of stay NOTECHS II	Controlled before-and-after study Data during admission 3 months after the implementation Pre-intervention *n* = 151 (26 observations) Post-intervention *n* = 136 (25 observations) Pre-control *n* = 418 (21 observations) Post-control *n* = 355 (24 observations)

NOTECHS II = nontechnical skills, measured using the Oxford NOTECHS II scale; TeamSTEPPS = Team Strategies and Tools to Enhance Performance and Patient Safety.

**Table 2:** Evidence table

Study reference	Study characteristics (i) Design (ii) Setting (iii) Country (iv) Funding	Patient or team characteristics (i) Inclusion criteria (ii) Exclusion criteria (iii) Number at baseline (iv) Important prognostic factors (v) Groups comparable at baseline?	Intervention (I)	Comparison/control (C)	Follow-up (i) Length of follow-up (ii) Loss to follow-up (iii) Incomplete outcome data	Outcome measures and effect size (i) Clinical (ii) Performance (iii) Costs
[21]	(i) Before-and-after study (ii) Total joint arthroplasty (iii) USA (iv) None	(i) NA (ii) NA (iii) NA (iv) NA (v) Unclear	High-efficiency OR: dedicated OR efficiency teams, parallel processing, dedicated hospital resources, modified physician behavior	Before implementation of the high-efficiency OR	(i) 3 years (ii) NA (iii) NA	**2. Performance** On-time OR starts 2007: <60% 2011: >90% Mean turnover times (mins) 2007: >60 2011: 35 Mean length of stay (days) Primary 2007: 3.5 2011: 3.1
[15]	(i) Retrospective cohort study (ii) Robotic-assisted sacrocolpopexy (iii) USA (iv) None	(i) All RASCs performed for pelvic organ prolapse from June 2010 to August 2015 performed by a single surgeon (ii) Surgeon performed cases without a fellow (iii) Intervention: 17 Control: 71 (iv) *Age ± SD:* I: 60.2 ± 8.65 C: 62.53 ± 10.76 BMI *± SD:* I: 28.71 (4.16) C: 28.45 (5.23) (v) Yes	A dedicated robotic team in the OR	No dedicated robotic team in the OR	(i) 5 years (ii) NA (iii) NA	**1. Clinical** Complications Intervention: 0% Control: 1.41%; *P* = 0.637 **2. Performance** Mean operative time (min) Intervention: 131.8 Control: 160.2 Length of stay, mean (days) Intervention: 1.29 Control: 1.20; *P* = 0.385 Readmission,*n*(%) Intervention: 0% Control: 4.4%; *P* = 0.545
[14] (included in review Sun, 2018)	(i) RCT (ii) Surgery (iii) France (iv) Hospital research grant	(i) NA (ii) NA (iii) Intervention: 8623 Control: 8222 (iv) *Age ± SD:* I: 57.9 (18.9) C: 59.1 (19.0) *Sex:* I: 41.4 % M C: 50.1% M (v) No	Team training in 16 hospitals. Two sessions at 6-month intervals. The first training session focused on CRM: situational awareness, team synergy and interpersonal communication. The second session focused on the implementation of checklists and CRM	No action in the 15 control hospitals	(i) In-hospital (ii) NA (iii) NA	**1. Clinical** Major adverse events: I: 8.8% to 5.5% (*P* < 0.001) C: 7.9% to 5.4% (*P* < 0.001) I: odds ratio 0.57, 95% CI 0.48 to 0.68; *P* < 0.001 C: odds ratio 0.64, 95% CI 0.50 to 0.81; *P* < 0.001 ROR: 0.90; *P* = 0.474, Inpatient mortality: ROR: 0.81; *P* = 0.581
[20]	(i) Controlled before–after study (ii) Elective orthopedic surgery (iii) USA (iv) None	(i) Posterior spinal fusion for scoliosis Category I and II patients: standard fusion and complex fusion (ii) Category III and IV patients: complex patient, complex fusion and other spine operations. Categories III and IV are extremely variable regarding anesthesia preparation time, surgical equipment needs, etc. (iii) Intervention: 78 Control: 89 (iv) *BMI ± SD:* *Category I* *I: 19.38 ± 2.01* *C:19.75 ± 2.33* *Category II* *I: 22.22 ± 4.59 C:21.96 ± 5.65* (v) Yes	Standardized protocols for anesthesia and perioperative patient management, patient transport, positioning, preparation, draping, imaging and wake-up were introduced in two phases. This included team training, parallel processes and weekly team communication (calendar). Phase 1: One core team with one surgeon. Phase 2: Scale-up with two surgeons	Before intervention (historical cases)	(i) 14 months (ii) NA (iii) NA	**1. Clinical** Complications: Intervention: 0% Control: 2.2% **2. Performance** Time saved by a dedicated team: Total room time (preoperative, operative time and postoperative time) time reduction (min): Category 1-phase 1: 104.9 (*P* < 0.01) Category 1-phase 2: 105.4 (*P* < 0.01) Category 1: 29.7% Category 2-phase 1: 75.8 (*P* < 0.01) Category 2-phase 2: 103.4 (*P* < 0.01) Category 2: 18.5% **3. Costs** Average cost reduction per case: Category 1: $8900 (*P* < 0.001) Category 2: $6000 (*P* < 0.001)
[12], (included in review Sun, 2018)	(i) Retrospective cohort study (ii) All surgeries (iii) USA (iv) None	(i) NA (ii) NA (iii) NA (iv) NA (v) NA	TeamSTEPPS program for all OR staff	Before training program implementation	(i) 9 months after intervention, 21 months after intervention (without support) (ii) NA (iii) NA	**1. Clinical** Mortality 9 months: 2.7% to 1%; *P* < 0.05 21 months: 1% to 1.5%; *P* < 0.05 Complications 9 months: 20.2% to 11.0%, *P* < 0.05 21 months: 11% to 13%; *P* < 0.05 **2. Performance** Turnover time 9 months: 43 to 35.5 min (*P* < 0.05). 21 months: decreased to 26.8 min OR staff teamwork 9 months: score 53.2 to 62.7 (scale unclear); *P* < 0.05 OR communication score 47. 5 to 62.7 (scale unclear); *P* < 0.05
[22]	(i) Prospective observational study (ii) Acute care (iii) USA (iv) None	(i) Patients with biliary tract disease admitted from 1 July 2005 to 30 June 2006, 2006 and from 1 July 2007 to 30 June 2009 the period 1 July 2006 to 30 June 2007 was omitted. (ii) Patients who were treated nonoperatively, had an elective cholecystectomy or had a primary etiology other than biliary tract disease. Incidental cholecystectomies were also excluded. (iii) 244 patients (iv) *Age:* I: 49.7 C: 51.2 *Sex:* *I*: 51.4 M C: 44.8% M (v) Yes	The ‘acute care surgery’ (ACS) model; comprised of nine dedicated surgeons who rotate weekly, as well as a senior resident, two junior residents and two hospitalists	Before implementation of the ACS model	(i) 2 years (ii) NA (iii) NA	**1. Performance** Mean time from admission/decision to OR in hours pre-ACS: 31.1 post-ACS: 21.5; *P* < 0.001 Mean operative time pre-ACS: 78.8 post-ACS: 98.9; *P* = 0.0013 Mean length of stay in days pre-ACS: 6.0 post-ACS: 6.0; NS Number of patients awaiting OR availability pre-ACS: 95.8% post-ACS: 60.7% *P* < 0.001 OR start time past daytime Hours pre-ACS: 29.2% post-ACS: 7% *P* < 0.001 OR start at night after waiting for daytime OR pre-ACS: 26.1% post-ACS: 7.9% *P* < 0.001
[25]	(i) Uncontrolled before-and-after study (ii) Cardiothoracic, neuro- and orthopedic surgery (iii) USA (iv) Unclear	(i) Surgical specialties from one center (ii) NA (iii) 50 ORs (iv) NA (v) NA	Development of interprofessional perioperative accountable teams through an educational program (based on the interprofessional education collaborative), team building, sharing information. A series of 1-day workshops was given	Before implementation	(i) 12 months (ii) NA (iii) NA	**2. Performance** Turnover time (min): Ortho: I: 75 C: 40 Neuro: I: 90 C:60 CTS: I: 75 C: 50 Average reduction of 30%. *(Exact numbers per specialty are displayed in figure only)* **3. Cost** Increase in revenue: >20% (*Exact numbers per specialty are displayed in figure only)*
[23]	(i) Controlled before–after studies (ii) Elective orthopedic, plastic or vascular surgery (iii) UK (iv) National Institute for Healthcare Research (NIHR) grant	(i) NA (ii) NA (iii) All interventions: 255 Control: 198 (iv) NA (v) NA	Three intervention strategies: (i) teamwork training based on aviation CRM (ii) standard operating procedures (iii) lean thinking	Comparisons before implementation and with control group: active-operating teams with no training	(i) 3 months (ii) NA (iii) NA	**1. Performance** Mean glitch count: Before: 7.21 After: 10.20 Difference: 2.99 (95% CI 1.16–4.82) NOTECHS II: Before: 71.62 After: 75.68 Difference: 4.06 (95% CI 0.93–7.20)
[19], (included in review Sun, 2018)	(i) Uncontrolled before–after study (ii) Laparoscopic cholecystectomy and carotid endarterectomy (iii) UK (iv) BUPA grant and Oxford hospitals research funding	(i) NA (ii) NA (iii) Before: 48 After: 55 (iv) *NA* (v) Unknown	A 9-h classroom nontechnical skills course based on aviation ‘Crew Resource Management’ (CRM) was offered to all staff, followed by 3 months of twice-weekly coaching from CRM experts	Before implementation	(i) 6 months (ii) NA (iii) NA	**1. Clinical** Complications Before: 5 After: 3 **2. Performance** Mean operating technical errors Before: 1.73 After: 0.98; *P* = 0.009 Mean operating time in minutes Before: 103.7 After: 101.2; *P* = 0.770 Mean length of stay (days) Before: 2.23 After: 2.02 *P* = 0.086 NOTECHS (SD) Before: 37.0 (4.3) After: 38.7 (3.2) *P* = 0.021
[17]	(i) Controlled interrupted time-series study (ii) Elective orthopedics and vascular/ general surgery (iii) UK (iv) NIHR grant	(i) NA (ii) NA (iii) Active before:26 Active after: 25 Control before: 11 Control after: 10 (iv) *Mean age* Active before: 57.8 Control before: 51.7 *Sex (% male):* Active before: 48% Control before: 52% (v) Unclear	Two 3-h sessions teamwork training course and communications training based on aviation CRM for all staff, followed by 6 weeks of weekly in-service coaching	No training (control) Active = before training	(i) 3 months after intervention period (ii) NA (iii) NA	**1. Clinical** Complications Active: 21.5% to 26.8% Control: 27.1% to 25.7%; *P* = 0.05 Readmission rate Active: 13% to 11% Control: 8.5% to 9%; *P* = 0.25 2**. Performance** Mean operation time in minutes Active: 113 to 115 Control: 98 to 71 Mean glitch rate: Active:7.21 to 10.20; *P* = 0.002 Control: 10.31 to 10.79; *P* = 0.796 Length of stay (days) Active: 11.1 to 13.2 Control: 13.5 to 11.7; *P* = 0.371 NOTECHS II score Active: 71.62 to 75.44 Control: 72.09 to 70.09; *P* = 0.047
[16]	(i) Controlled interrupted time-series study (ii) Elective orthopedic (iii) UK (iv) NIHR grant	(i) NA (ii) NA (iii) Pre-intervention: 567 Post-intervention: 679 Pre-control: 544 Post-control: 421 (iv) *Unknown* (v) Unknown	SOPs and team training, morning briefing and conduct of World Health Organization (WHO) Surgical Safety Checklist 1-day interactive lecture-based training, using aviation CRM training	No intervention	(i) 6 months (ii) NA (iii) NA	**1. Clinical** Complication rate Intervention: 14 to 18% Control: 11 to 15%; *P* = 0.33 Readmission rate Intervention: 13 to 11% Control: 20 to 20%; *P* = 0.29 **2. Performance** Mean glitch rate: Intervention: 6.84 to 5.25 Control: 7.67 to 6.52; *P* = 0.153 Length of hospital stay (days) Intervention: 11 to 7.2 Control: 19 to 20; *P* = 0.376 NOTECH II Intervention: 74.83 to 79.27 Control: 76 to 71.81; *P* = 0.002
[13]	(i) Retrospective study with control group (ii) Noncardiac surgery (iii) USA (iv) Veterans health national center grant	(i) Operations under general, spinal or epidural anesthesia, noncardiac (ii) Procedures with known low morbidity and mortality or transurethral resections of the prostate or bladder and herniorraphies >5/week (iii) Intervention: 74 facilities Control: 34 facilities (iv) NA (v) Unclear	Veterans Health Administration (VHA) Medical Team Training program	Not (yet) trained	(i) 1 year (ii) NA (iii) NA	**1. Clinical** 30-day mortality: Intervention: −18% RR: 0.82; (95% CI, 0.76–0.91); *P* = 0.01 Control: −7% RR, 0.93; (95% CI, 0.80–1.06); *P* = 0.59 Dose–response relationship: for every quarter of the training program, a reduction of 0.5 deaths per 1000 procedures occurred (95% CI, 0.2–1.0; *P* = 0.001)
[24]	(i) Before–after study (ii) Robot-assisted laparoscopic radical prostatectomy (iii) USA (iv) Unclear	(i) Before: August 2008–January 2009 After: April–November 2009 (ii) NA (iii) Before:100 After: 100 (iv) *Age (range)* Before: 61.1 (47–76) After: 59.4 (41–77) *BMI (range):* Before: 27.6 (18.3–50.2) After: 27.6 (19.5–39.4) (v) Yes	Four modifications: adherence to time-oriented surgical goals; dedicated anesthesia team; simultaneous processing by nursing and urology house staff during case turnover; identification and elimination of unused disposable instruments	Before intervention	(i) I: 8 months C: 6 months (ii) NA (iii) NA	**1. Performance** Mean surgical time, min Before: 256.2 After: 238.8 Difference: −7.4 (−6.8%); *P* = 0.04 Mean anesthetic time, min Before: 23.7 After: 19.2 Difference: −4.5 (−19.1%); *P* = 0.006 Mean turnover time, min Before: 43.0 After: 30.9 Difference: −12.1 (−28.1%); *P* = 0.005 Total costs Before: 11 806 After: 29 Difference (% change): −2548 (−21.7%)
[18]	(i) Controlled before–after study (ii) Elective orthopedic and reconstructive surgery (iii) UK (iv) NIHR grant	(i) NA (ii) NA (iii) Before active group: 151 After active group: 136Before control: 418 After control: 355 (iv) NA (v) Different setting, but similar skill mix	Combining teamwork training spread over 6 weeks, focusing on WHO surgical safety checklist process, and other nontechnical skills. AND lean process improvement	No intervention in control group (or before intervention)	(i) 3-month intervention with 3 months data collection period before and after. (ii) NA (iii) NA	**1. Clinical** Complication rate Active: 9 to 7% Control: 18 to 28%; *P* = 0.08 Readmission rate Active: 2% to 9% Control: 1% to 2%; *P* = 0.33 **2. Performance** Mean operating time Active: 2 h 17 min Control: 1 h 36 min; NS Glitch rate Active: 10.48 to 4.38 Control: 9.79 to: 13.20; *P* < 0.001 Mean length of stay (days) Active: 6.3 to 4.0 Control: 7.2 to 7.9; *P* = 0.09 NOTECHS II Active: 69.81 to 75.56 Control: 72.88 to 72.54; *P* = 0.058

BMI = Body mass index; LRYGB = Laparoscopic Roux-en-Y gastric bypasses; NA = not applicable; NOTECHS II = nontechnical skills, measured using the Oxford NOTECHS II scale; TeamSTEPPS = Team Strategies and Tools to Enhance Performance and Patient Safety; SD = standard deviation; CI = Confidence Interval; ROR = Reporting Odds Ratio; RR = Relative Risk.

Assessment of the risk of bias of the individual studies is shown in Tables 3 and 4. Overall, the risk of bias was high.

**Table 3:** Risk of bias table for intervention studies (RCTs)

Study reference (first author, publication year)	Describe the method of randomization	Bias due to inadequate concealment of allocation? (unlikely/likely/unclear)	Bias due to inadequate blinding of participants to treatment allocation? (unlikely/likely/unclear)	Bias due to inadequate blinding of care providers to treatment allocation? (unlikely/likely/unclear)	Bias due to inadequate blinding of outcome assessors to treatment allocation? (unlikely/likely/unclear)	Bias due to selective outcome reporting on basis of the results? (unlikely/likely/unclear)	Bias due to loss to follow-up? (unlikely/likely/unclear)	Bias due to violation of intention to treat analysis? (unlikely/likely/unclear)
[14]	Participating hospitals were randomized into two cluster arms (on hospital level). To ensure comparability between arms, randomization was balanced across geographical areas. Additionally, hospitals were allocated according to major adverse events collected during the pre-implementation period to guarantee similar primary outcomes between arms at baseline. Every month in each participating hospital, the first 50 adults admitted for surgical procedures were eligible for inclusion	Unlikely	Unclear	Likely	Likely	Unlikely	Unlikely	Unlikely

**Table 4:** Risk of bias table for intervention studies (observational: non-randomized clinical trials, cohort and case–control studies)

Study reference (first author, year of publication)	Bias due to a nonrepresentative or ill-defined sample of patients?	Bias due to insufficiently long, or incomplete follow-up, or differences in follow-up between treatment groups?	Bias due to ill-defined or inadequately measured outcome?	Bias due to inadequate adjustment for all important prognostic factors?
[21]	Unclear	Unlikely	Unlikely Likely for observed outcome measures (not blinded)	Likely
[15]	Unlikely Possible selection bias in dedicated team group, however they used a single-surgeon database	Unlikely However, large different in number of observed patients between groups	Unlikely	Unlikely (a multiregression model was provided for operating time, not for other outcome variables, however, as there were few cases adjustments would not be relevant) No adjustment for other factors that relate to time efficiency e.g. OR staff turnover or hospital-driven quality control measures
[22]	Unclear (uncontrolled before–after study)	Unlikely	Unlikely Likely for observed outcome measures (not blinded)	Likely
[25]	Unclear (uncontrolled before–after study)	Unlikely	Unlikely	Likely
[23]	Unlikely (minimal contamination)	Unlikely	Unlikely Likely for observed outcome measures (not blinded)	Likely
[19]	Unclear (uncontrolled before–after study)	Unlikely	Unlikely Likely for observed outcome measures (not blinded)	Likely
[20]	Unlikely Possible selection bias in dedicated team group over time (4 years later), however they used the same surgeons	Unlikely	Unlikely	Likely
[12]	Unclear (uncontrolled before–after study)	Unlikely	Unlikely Likely for observed outcome measures (not blinded) Teamwork outcomes are unclear	Likely
[14]	Unlikely (cluster RCT)	Unlikely	Unlikely	Unlikely (cluster RCT)
[17]	Unlikely	Unlikely	Unlikely Likely for observed outcome measures (not blinded)	Likely
[16]	Unclear	Unlikely	Unlikely Likely for observed outcome measures (not blinded)	Likely
[13]	Likely	Unlikely	Unlikely	Unlikely (propensity matching)
[24]	Unlikely	Unlikely	Unlikely Likely for observed outcome measures (not blinded)	Likely
[18]	Unlikely	Unlikely	Unlikely Likely for observed outcome measures (not blinded)	Likely

#### Clinical outcomes

##### Mortality

Mortality was assessed in two NRSs and one cluster RCT [12–14]. Team training was provided in all three studies (see Table 3 for details). Neily *et al.* found a reduction in mortality for noncardiac surgery in both intervention (almost 50%) and control groups [13]. However, the reduction was significantly larger in the intervention group (propensity matching: adjusted RR: 1.49; 95% CI 1.10–2.07). A dose–response relationship was observed quarterly during the training program, with a reduction of 0.5 deaths per 1000 operations (95% CI 0.2–1.0).

Forse *et al*. found a significant reduction in mortality from 2.7% to 1% (*P* < 0.05) 9 months after the intervention in their retrospective cohort [12]. One year later, however, this had increased to 1.5%. Duclos *et al.* also observed a significant decrease in mortality in both intervention and control groups in their cluster RCT [14]. Nonetheless, there was no significant difference between the intervention and control groups, attributed to flawed methodology. Likely, there was bias due to inadequate blinding of care providers and outcome assessors (ROR 0.81, 95% CI 0.38–1.72).

##### Complications

Eight studies evaluated a wide range of reported complications [12, 14–20]. In two smaller studies without control groups, few complications were recorded, and no difference was found before and after implementing the intervention. The subject of the studies was a dedicated robotic team who underwent CRM training plus coaching [15, 19].

In three NRSs, fewer complications were seen in the intervention group, but this difference did not reach statistical significance [16, 18, 20]. Comparing the primary outcome between these studies was difficult, as the setting and type of patients in these studies differed. In a large retrospective cohort study, a significant decrease in morbidity was observed 9 months after the implementation of the intervention (TeamSTEPPS program). Complications decreased from 20% to 11% (*P* < 0.05). After 1 year, a relapse rate was observed with an increase in complications from 11% to 13% (*P* < 0.05) [12].

In a cluster RCT, a significant decrease in complications was seen in both groups (from 8.8% to 5.5% in the intervention group and from 7.9% to 5.4% in the control group) [14]. Nevertheless, there was no difference between the intervention and control groups (ROR 0.90, 95% CI 0.67–1.21). No differences were seen in major complications or postoperative complications. In contrast, in another controlled before-and-after study a slight, yet significant, increase in complications in the intervention group (21.5% to 26.8%), attributed to an increased glitch count, and a slight decrease in the control group (27.1% to 25.7%) were noted [17].

##### Readmissions

Four studies looked at readmission rates [15–18]. None of these found a significant difference in readmission rates between the intervention (treated by dedicated team) and control groups over time.

#### Performance outcomes

##### Efficiency

###### Length of hospital stay

Seven studies reported on length of hospital stay [15–19, 21, 22]. For this outcome measurement, the treatment by a dedicated team made no difference.

###### Glitch count

No study was able to show a reduction in glitch count for dedicated teams [16, 17, 23]. Nonetheless, in dedicated teams, the amount of technical operative mistakes and nonoperative procedural mistakes was lower than that in non-dedicated teams [23]. These outcomes are however not comparable to glitch count.

###### Surgery duration and turnover time

There are conflicting results regarding the effect of a dedicated team on surgery duration. Four studies found reductions in surgery time when using a dedicated team, ranging from 7% to 30% reduction in surgery time and a 5% reduction in anesthesiology time [15, 19, 20, 24].

In contrast, two studies saw an increase in surgery time [17, 22]. Morgan *et al.* saw a small non-significant increase, and Lim *et al.* found an increase in surgery duration in the acute setting; however, the time from admission until decision to operate was significantly shorter [17, 22].

Four studies reported on turnover time, all finding significant reductions in turnover time, ranging between 28% and 41% [12, 21, 24, 25].

##### Teamwork and communication

###### Oxford NOTECHS II

Improvements in Oxford NOTECHS II (a rating system for nontechnical skills of a surgical team) over time were observed in all studies, with most improvement seen in the dedicated teams [16–19, 23]. The improvement of nontechnical skills was only significant for nurses or for anesthesiologists, but never for surgeons [16–18].

###### Teamwork and team communication

Team climate and teamwork improved in a dedicated team, as assessed by the Safety Attitudes Questionnaire (SAQ) and a program-specific questionnaire [12, 19]. A non-significant improvement of team communication after 9 months was reported by the dedicated surgical team, assessed by a program-specific questionnaire [12].

#### Costs

Three studies evaluated cost outcomes [20, 24, 25]. Two studies found statistically significant reductions in cost [20, 24]. Flynn *et al.* reported an average cost reduction of $8900 and $6000 for more complex cases [20]. Rebuck *et al.* noted that anesthesiology-related costs remained the same, but total costs decreased with 22% [24]. One study from the USA reported a 20% increase in OR revenue, attributed to increased efficiency [25].

## Discussion

### Statement of principal findings

This systematic literature review is the first to evaluate the effects of a dedicated surgical team on clinical outcomes and performance measures. Effective implementation of a dedicated team is often assumed to be advantageous, but in clinical practice sometimes difficult to realize. This review provides an analysis of benefits for the development and implementation of dedicated teams. Implementation of dedicated surgical teams was found to be associated with improvements in several outcomes, including mortality, turnover time, nontechnical skills, teamwork, team communication and costs. No significant differences were observed in readmission rates and length of hospital stay. The effect of a dedicated surgical team on operation time, disturbances and complications remains unclear, due to inconsistent findings.

### Interpretation within the context of the wider literature

The improvements in outcomes following the implementation of a dedicated surgical team are in agreement with literature regarding the positive effects of surgical team training [1, 8].

An overall decrease in mortality was observed, when studying surgical teams over time. Whether the formation of a formal dedicated team adds to this remains controversial [12–14]. Follow-up time, intervention type and frequency at which trainings were given, was inconsistent across studies. The effect of a dedicated surgical team on complications remains unclear as results were inconsistent across studies. A wide range of complications was investigated, making it difficult to compare and draw conclusions [12, 14–20].

Results regarding glitch counts were contradictive. While in most studies, fewer technical operative mistakes and nonoperative procedural mistakes were reported in the dedicated team, in one RCT more glitches were observed; the reason behind the worsened glitch counts remains unclear [16, 17, 23]. The effect of dedicated surgical teams on surgery duration remains unclear, due to inconsistent results [15, 17, 19, 20, 22, 24]. One study attributed increased surgery duration to more teaching taking place during surgeries [22]. However, most studies found the effect of dedicated teams on surgery duration to be beneficial [15, 19, 20, 24]. Turnover time and costs were found to be significantly reduced by implementing a dedicated surgical team [12, 20, 21, 24, 25]. Cost reductions were attributed to increased efficiency and reduced turnover time, and hence, reductions in wages, surgical equipment and use of surgical theaters [20, 24]. In line with this finding, teamwork improved [12, 21, 24]. In four out of five studies, significant differences existed in nontechnical skills between intervention and control groups [16–19, 23]. In accordance, the implementation of a dedicated surgical team was associated with a significant improvement in teamwork [12, 19]. Although team communication was also found to improve after the intervention, this did not reach statistical significance [12].

### Strengths and limitations

The strengths of this review include the comprehensive search strategy developed by an information specialist and discussion between reviewers in case of doubt. Moreover, a wide array of outcomes and settings was considered to evaluate dedicated surgical teams.

The level of evidence regarding clinical, efficiency, teamwork, communication and cost outcomes is limited because of study conduct. Most studies were observational studies with little to partial correction for confounders, leading to inconsistent results. This review is limited by the quality and the data of the included studies. Most studies had some significant drawbacks in study design and a large variety of interventions, settings and recorded data, making it hard to compare and draw conclusions.

A fundamental limitation is the lack of a clear definition of what a dedicated team entails and how such a team should be formed. As a result, studies have interpreted a dedicated team in various manners. The formation of a dedicated team varied across studies; from teams receiving little trainings to teams receiving frequent trainings over the span of months. Furthermore, not all teams received technical and communicative team training; in an ideal scenario this would have been the case. Whether there can be a universal definition of a dedicated team is unclear; surely what ‘dedicated’ entails varies per specialism and setting. We propose that a surgical team is everybody in the OR with a role in the care for the patient. The team becomes dedicated when these individuals train together in technical and nontechnical skills. Attention should be paid to the frequency and continuity of training sessions as dose–response and relapse rates were seen. Furthermore, a standardized set of core outcomes could help overcome problems in measuring the effects of a dedicated team.

Moreover, there is no ideal outcome to measure the effect of the intervention. When considering clinical outcomes, mortality and complications are confounded by many factors. Performance outcomes are also confounded by several factors, making it difficult to draw conclusions. Glitch count is used as a proxy for surgical efficiency, but what exactly entails a ‘glitch’ remains unclear and may differ between studies. The cost–benefits of implementing a dedicated surgical team are interlinked with surgical efficiency; one could say that a more efficient surgery is also cheaper.

### Implications for policy, practice and research

The main implication of this review is that a dedicated surgical team, through team training, may contribute to the optimization of healthcare quality and patient safety within the perioperative period. Through team training, technical and nontechnical skills may improve, increasing the level of teamwork and effectiveness of communication. This leads to better efficiency of the operative and perioperative processes, reducing costs. Moreover, this reduces mistakes and mortality. The sustainability of implementing a dedicated surgical team needs to be further investigated, as only a few studies have evaluated this. A dose–response effect of team training and a relapse rate were reported [12, 13]. This is in-line with findings in other studies regarding nontechnical skills training for surgical teams [4].

The implications of these results have been assimilated into a recommendation in the Dutch perioperative guideline [26].

The generalizability of these results also needs to be further investigated. This review mainly looked at complex procedures. One could hypothesize that dedicated surgical teams yield most benefits during highly specialized and routine surgeries. In highly complex interventions, the mismatch between highly specialized surgeons and nonspecialized team members is the greatest, suggesting that dedicated teams could provide most benefits in this setting.

## Conclusion

This systematic review summarizes the potential benefits of dedicated surgical teams in the perioperative setting. It seems to be beneficial to offer team training to improve technical and/or nontechnical skills of surgical teams. Dedicated teams could help optimize healthcare and increase comprehension of each other’s actions [27, 28].

However, further studies are needed to draw conclusions, as the level of evidence was often low. Standardized team training, settings and methods of recording outcomes, for both technical and nontechnical skills, could help in comparing studies and group results.

## Supplementary Material

mzac078_Supp

## Data Availability

As this is a systematic review, all data relevant to the study are included in the article, see methods and evidence tables. If further information is needed, this can be requested from f.willeboordse@kennisinstituut.nl.
