# Supplementary material for: Dedicated teams to optimize quality and safety of surgery: A systematic review
Source: Int J Qual Health Care. 2022 Oct 6;34(4):mzac078. doi: 10.1093/intqhc/mzac078 (PMC9606443; doi:10.1093/intqhc/mzac078)
Supplement: mzac078_Supp [file mzac078_supp.zip › suppl_data/Supplementary_Data.docx]

**SUPPLEMENTARY MATERIAL**

**Supplementary file 1**

**Pubmed and Embase Search strategy**

Database: Medline (OVID)

Search terms:

1: exp *General Surgery/ or surger*.ti. or surgic*.ti. or operat*.ti. or peroperat*.ti. or preoperat*.ti. or perioperat*.ti. or postoperat*.ti. or intraoperat*.ti. (804645)

2: exp Patient Safety/ or exp Work performance/ or exp Communication/ or exp Intraoperative complications/ or exp Surgical wound infection/ or exp Postoperative complications/ or exp performance.ti,ab. or communication.ti,ab. or adverse event*.ti,ab. or error*.ti,ab. or mistake*.ti,ab. or complication*.ti,ab. or safety.ti,ab. or quality.ti,ab. or efficiency.ti,ab. or site infection*.ti,ab. or flow disruption*.ti,ab. or compliance.ti,ab. (3917496)

3: exp *Cooperative Behavior/ or ((dedicat* or specialize* or specialise* or efficiency) adj3 team*).ti,ab. or specialist team*.ti. or teamwork.ti. or team work.ti. (20676)

4: 1 and 2 and 3 (495)

5: limit 4 to (English language and yr=”2000-Current”) (360)

**Supplementary file 2**

**Prisma flowchart**

**
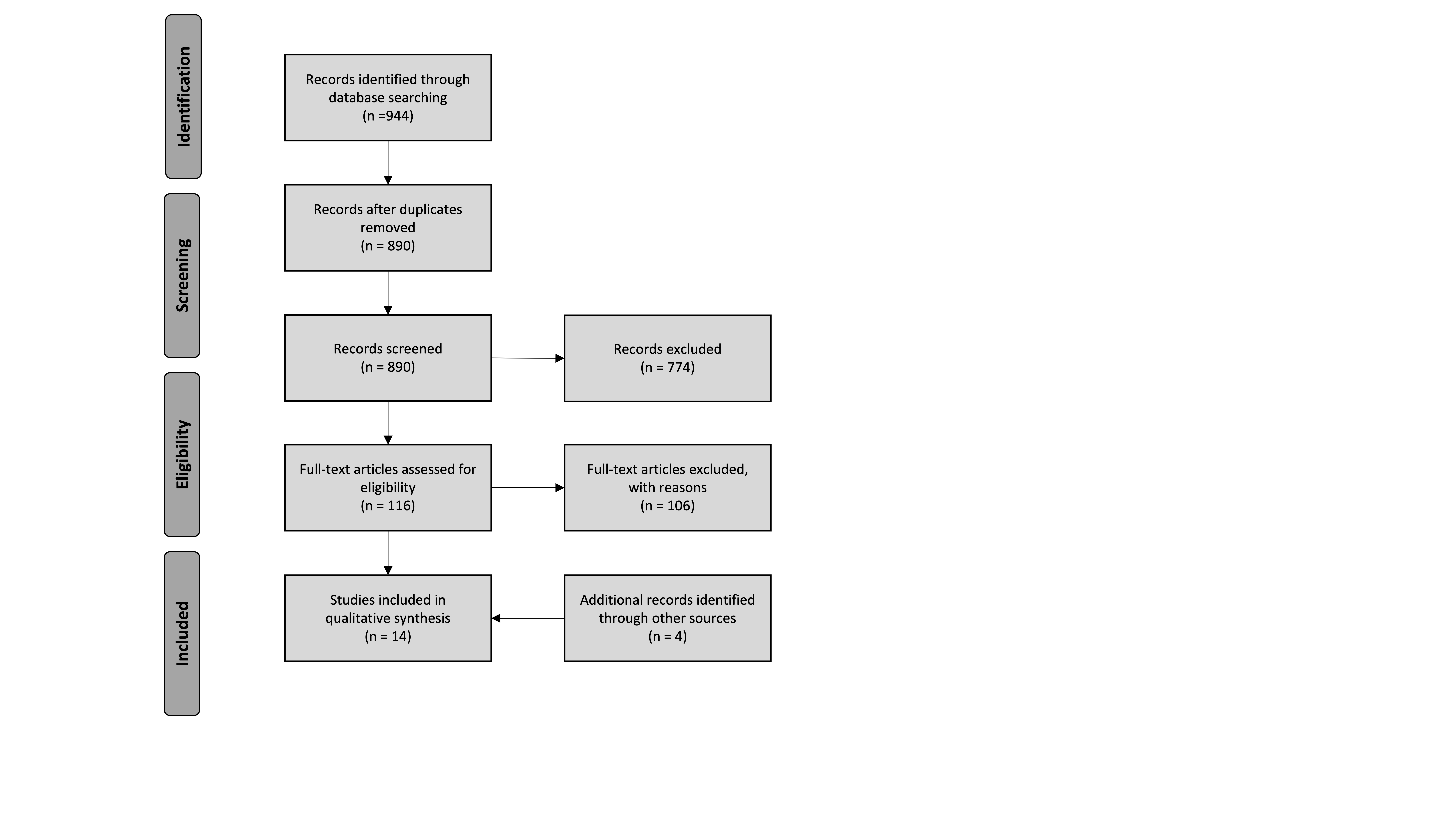
**

**Supplementary file 3**

**PICOS criteria**

PICO question

**P (Patients):** All patients undergoing surgery.

**I (Intervention)**: Dedicated operating team.

**C (Comparison):** No dedicated operating team.

**O (Outcomes)**: 1. Clinical outcomes (morbidity, mortality, other adverse events); 2. Performance outcomes (surgery duration, delays, length of hospital stay, readmissions); 3. Teamwork and communication; 4. Costs.

| **Criteria** | **Include** | **Exclude** |
| --- | --- | --- |
| Date | Studies conducted after 2000 | Studies conducted before 2000 |
| Study design | Randomized control trials or non-randomized comparative studies | All other study designs |
| Location | Studies conducted in the Western world | Studies conducted in non-Western countries |
| Sample size | A sample size of at least 20 surgeries | A sample size smaller than 20 surgeries |
| Outcomes | Studies reporting at least one clinical outcome parameter | Studies not reporting any clinical outcome parameters |

**Supplementary file 4**

**PRISMA checklist**

| **Section/topic** | | **#** | **Checklist item** | | | **Reported on page #** |
| --- | --- | --- | --- | --- | --- | --- |
| **TITLE** | | | | | |  |
| Title | | 1 | Identify the report as a systematic review, meta-analysis, or both. | | | 1 |
| **ABSTRACT** | | | | | |  |
| Structured summary | | 2 | Provide a structured summary including, as applicable: background; objectives; data sources; study eligibility criteria, participants, and interventions; study appraisal and synthesis methods; results; limitations; conclusions and implications of key findings; systematic review registration number. | | | 2 |
| **INTRODUCTION** | | | | | |  |
| Rationale | | 3 | Describe the rationale for the review in the context of what is already known. | | | 4 |
| Objectives | | 4 | Provide an explicit statement of questions being addressed with reference to participants, interventions, comparisons, outcomes, and study design (PICOS). | | | 4 |
| **METHODS** | | | | | |  |
| Protocol and registration | | 5 | Indicate if a review protocol exists, if and where it can be accessed (e.g., Web address), and, if available, provide registration information including registration number. | | | 2 |
| Eligibility criteria | | 6 | Specify study characteristics (e.g., PICOS, length of follow-up) and report characteristics (e.g., years considered, language, publication status) used as criteria for eligibility, giving rationale. | | | 5 |
| Information sources | | 7 | Describe all information sources (e.g., databases with dates of coverage, contact with study authors to identify additional studies) in the search and date last searched. | | | 5 |
| Search | | 8 | Present full electronic search strategy for at least one database, including any limits used, such that it could be repeated. | | | Supplementary file |
| Study selection | | 9 | State the process for selecting studies (i.e., screening, eligibility, included in systematic review, and, if applicable, included in the meta-analysis). | | | 5 |
| Data collection process | | 10 | Describe method of data extraction from reports (e.g., piloted forms, independently, in duplicate) and any processes for obtaining and confirming data from investigators. | | | 6 |
| Data items | | 11 | List and define all variables for which data were sought (e.g., PICOS, funding sources) and any assumptions and simplifications made. | | | 6 |
| Risk of bias in individual studies | | 12 | Describe methods used for assessing risk of bias of individual studies (including specification of whether this was done at the study or outcome level), and how this information is to be used in any data synthesis. | | | 6 |
| Summary measures | | 13 | State the principal summary measures (e.g., risk ratio, difference in means). | | | 6 |
| Synthesis of results | | 14 | Describe the methods of handling data and combining results of studies, if done, including measures of consistency (e.g., I^2^) for each meta-analysis. | | | 7 |
| Risk of bias across studies | 15 | | | Specify any assessment of risk of bias that may affect the cumulative evidence (e.g., publication bias, selective reporting within studies). | 7 | |
| Additional analyses | 16 | | | Describe methods of additional analyses (e.g., sensitivity or subgroup analyses, meta-regression), if done, indicating which were pre-specified. | NA | |
| **RESULTS** | | | | |  | |
| Study selection | 17 | | | Give numbers of studies screened, assessed for eligibility, and included in the review, with reasons for exclusions at each stage, ideally with a flow diagram. | 6 | |
| Study characteristics | 18 | | | For each study, present characteristics for which data were extracted (e.g., study size, PICOS, follow-up period) and provide the citations. | 7 | |
| Risk of bias within studies | 19 | | | Present data on risk of bias of each study and, if available, any outcome level assessment (see item 12). | 18 | |
| Results of individual studies | 20 | | | For all outcomes considered (benefits or harms), present, for each study: (a) simple summary data for each intervention group (b) effect estimates and confidence intervals, ideally with a forest plot. | 9 | |
| Synthesis of results | 21 | | | Present results of each meta-analysis done, including confidence intervals and measures of consistency. | 22 | |
| Risk of bias across studies | 22 | | | Present results of any assessment of risk of bias across studies (see Item 15). | 18 | |
| Additional analysis | 23 | | | Give results of additional analyses, if done (e.g., sensitivity or subgroup analyses, meta-regression [see Item 16]). | NA | |

| **DISCUSSION** | | |  |
| --- | --- | --- | --- |
| Summary of evidence | 24 | Summarize the main findings including the strength of evidence for each main outcome; consider their relevance to key groups (e.g., healthcare providers, users, and policy makers). | 24 |
| Limitations | 25 | Discuss limitations at study and outcome level (e.g., risk of bias), and at review-level (e.g., incomplete retrieval of identified research, reporting bias). | 25 |
| Conclusions | 26 | Provide a general interpretation of the results in the context of other evidence, and implications for future research. | 26 |
| **FUNDING** | | |  |
| Funding | 27 | Describe sources of funding for the systematic review and other support (e.g., supply of data); role of funders for the systematic review. | 1 |

*From:*  Moher D, Liberati A, Tetzlaff J, Altman DG, The PRISMA Group (2009). Preferred Reporting Items for Systematic

Reviews and Meta-Analyses: The PRISMA Statement. PLoS Med 6(7): e1000097. doi:10.1371/journal.pmed1000097

For more information, visit: **www.prisma-statement.org**.
